# Supplementary material for: The Effect of Voriconazole on Tacrolimus in Kidney Transplantation Recipients: A Real-World Study
Source: Pharmaceutics. 2022 Dec 7;14(12):2739. doi: 10.3390/pharmaceutics14122739 (PMC9785881; doi:10.3390/pharmaceutics14122739)
Supplement: Supplementary file 1 [file pharmaceutics-14-02739-s001.zip › pharmaceutics-2034548-supplementary.pdf]

TableS1 The independent influencing factors of C_{Tac}

Parameters	Estimate Coefficients	Std.Error	t	VIF	*P*
(Intercept)	0.434	1.250	0.347		0.729
CYP3A5*3*3	0.604	0.243	2.482	1.515	0.013
Daily Dose	0.400	0.060	6.647	2.718	<0.001
CVRC	0.651	0.106	6.128	2.408	<0.001
NEUT	0.029	0.008	3.623	1.290	<0.001
PLT	0.003	0.002	2.066	1.454	0.039
ALT	0.015	0.008	1.979	5.225	0.048
AST	-0.034	0.013	-2.656	5.315	0.008
TBIL	0.121	0.053	2.292	5.062	0.022
ALB	0.053	0.025	2.103	1.195	0.036
BUN	0.022	0.010	2.120	2.257	0.034
CCR	0.018	0.006	3.108	2.386	0.002
Age	-0.025	0.012	-2.020	1.420	0.044
CYP2C19 substrate	-3.710	0.728	-5.098	14.372	<0.001
CYP2C19 inhibitor	3.641	0.651	5.592	13.021	<0.001
CYP3A4 substrate	-0.503	0.194	-2.598	4.447	0.009
CYP3A4 inhibitor	0.999	0.234	4.264	2.782	<0.001
CYP1A2 inducer	0.824	0.292	2.820	2.703	0.005
Inducer CYP2E1	-7.514	1.849	-4.065	1.215	<0.001
CYP2C9 substrate	-0.538	0.205	2.622	1.621	0.009
CYP2D6 substrate	-0.544	0.248	-2.191	1.339	0.029
F			10.29		
R^2			0.1857		
Ajusted R^2			0.1676		
P			<0.001		

Multiple linear regression was performed using stepwise method. The standard for inclusion was 0.05; while the standard for exclusion was 0.10 (N=1701).

TableS2 The independent influencing factors of CDW

Parameters	Estimate Coefficients	Std.Error	t	VIF	*P*
(Intercept)	0.075	0.025	3.032		0.002
Sex (male)	0.023	0.008	3.063	1.337	0.002
CYP3A5*3*3	0.026	0.006	4.062	1.839	<0.001
Daily Dose	-0.011	0.001	-7.849	1.292	<0.001
C_{VRC}	0.044	0.002	18.184	1.350	<0.001
NEUT	0.000	0.000	2.042	2.214	0.041
BUN	0.001	0.000	3.512	2.250	<0.001
CCR	0.000	0.000	2.683	1.493	0.007
WT	-0.001	0.000	-4.965	1.114	<0.001
Metabolic type of CYP3A4	-0.006	0.003	-2.206	1.337	0.028
F			101.4		
R2			0.5161		
Ajusted R^2			0.511		
P			<0.001		

Multiple linear regression was performed using stepwise method. The standard for inclusion was 0.05; while the standard for exclusion was 0.10 (N=1701).
